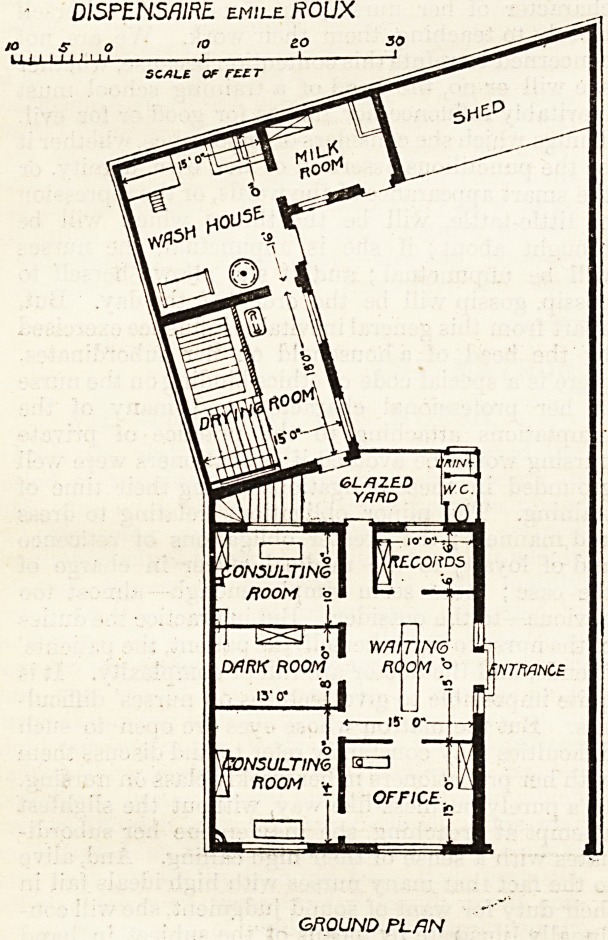# The "Emile Roux" Dispensary at Lille

**Published:** 1905-09-30

**Authors:** 


### THE "EMILE ROUX" DISPENSARY AT LILLE.

The institution whose name appears at the head of this article is an important factor in the active warfare now being carried on in France with tuberculous disease, and inasmuch as no exactly similar organisation has been set on foot in this country a description of its work may not be unprofitable.

The dispensary is not, in the ordinary sense of the word, a charitable institution for the relief of the sick by giving either advice or medicine; its rôle is rather "to seek out, to attract and to retain, by intelligent propaganda made in populous centres, workmen attacked or suspected of tuberculosis; to give them as often and as long as they need it, advice for themselves and their families; to distribute to them when they are compelled to suspend work, relief in food, clothes, bedding, spittoons, antiseptics, to purify their habitations by frequent cleansings and, disinfection repeated at regular intervals; to get for them, if needs be, a more healthy dwelling-place; to wash gratuitously their linen in order to avoid contagion in and outside the family; to make all useful applications of private benevolence; and to obtain help which will permit of the recovery of the patient if he is not too seriously attacked, and to send him back to his work." It was with such a task in view that the promoters of the dispensary "Emile Roux" at Lille set about their work.

The town of Lille has a population of 220,000; nearly 6,000 of this total are tuberculous poor, of whom 1,000 to 1,200 die annually, after having, as is pointed out by the promoters, sown all around them contagion and misery.

The work of the dispensary was begun in a room in the Pasteur Institute in February 1901, and was established in its permanent home in December of the same year. The main building, which abuts on the street (Boulevard Louis XIV.)

DISPENSAIRE EMILE ROUX

GROUND PLAN

contains a waiting-room for patients, two consultation-rooms, one of which is also the medical director's office, an office for the workman inquirer (an important official whose functions we shall describe later), a dark room for laryngoscope work, a room where ordinarily the registers are kept and the measurements of patients taken, and which in case of need can serve as a third consultation-room. The building at the rear of the main block contains a wash-house and drying-room and a room for the distribution of milk. The two blocks are joined by a glass roof open at the sides, and in the covered yard thus formed are a w.c. and urinal.

The medical council of the dispensary comprises all doctors who agree to give their services gratuitously to the work. The permanent staff consists of a medical director charged with the organisation of the work and the supervision of the assistants, three medical assistants whose work is the ordinary consultations. Laryngoscopic examinations are made by Professor Gaudier either on a fixed day or in the course of his ordinary work at the Hospital St. Sauveur.

Of the lay staff the workman inquirer is the most importan member. His function is to make domiciliary visits at the houses of the poor in the capacity of a sympathetic comrade, to converse in a friendly way with the parents of a patient, to inquire into their needs, their means of livelihood, and their habits; from the report which he will render the director will decide whether, and in what form, aid should be given in each case; and the workman inquirer will visit each case periodically and repeat in homely language the directions which are laid down for the guidance of patients and their friends. He will, for example, explain the use of such things as spittoons for table and pocket use. He will teach the use of antiseptics, and explain the kind of food most suitable for the patient and the proper way of preparing it, and he will give advice on the proper care of the body, sleep, and exercise. It is found in practice that advice thus imparted by a fellow-workman and in homely terms is not only better understood, but more readily followed, than if it were given by visitors of a class socially distant from the visited.

Two women are employed in the care generally of the premises and in the laundry, and a male assistant has charge of the distribution of milk and sees to the cleansing of the houses or lodgings of patients.

When a new patient whose name has been duly inscribed on the register presents himself at the dispensary, he is examined by one of the physicians who compiles his *dossier clinique* or clinical notes. These comprise:—(1) The study of the patients' antecedents hereditary and personal; (2) His chest measurement; (3) Height and weight and other physical details; (4) Examination and graphic record of pulmonary lesions; (5) Examination of circulation, digestive organs, the action of the kidneys and of the skin; (6) If necessary, examination of the larynx.

If the patient proves to be tuberculous, or there is a suspicion of his being so, the physician proceeds to instruct him on the main principles of hygiene with such special teaching as he deems most urgent—he tries to make him understand that it is to his own interest to follow scrupulously the advice he has listened to; he fixes the date for the next attendance, and supplies the patient with a pocket and a table spittoon, a bottle containing a 2 per cent. solution of lysol, and simple printed instructions drawn up in the homeliest way embodying all the essential points of the advice the patient has just listened to.

Following on this come the visits of the workman inspector which we have described above.

The average daily attendance is 120; of this number 50 or 60 at least have their linen disinfected and washed. Considerable importance is attached to this part of the work in view of the danger which results from the washing of clothes in the one living room, in the midst of children, and often in the very presence of the patient. In certain cases the patients are provided with bedding, sometimes also with beds which remain the property of the dispensary but are never reclaimed during the life of the patient. On the death of a patient the things are removed by the municipal sanitary authority, disinfected, and returned to the dispensary.

To sum up the objects which the promoters of this institution have set before them: the education of those who live in the district where the disease abounds in the laws of health; teaching the people how they can and ought to guard themselves and those dependent on them from propagation of the disease before the attack, and at the first sign of attack to arrest as far as possible the spread of the disease before it is too late.

Writing a year after the opening of the Lille Dispensary, Dr. Calmette, Directeur de l'Institut Pasteur, Dr. Verhaege, Medical Director, and M. Th. Waehrel, Administrator of the Dispensary, say:—"It is no longer possible to doubt the success of this propaganda. . . . The Emile Roux Dispensary represents the most simple, the most economical, and at the same time the most efficacious instrument which can be imagined."

Since its inception the scope of the work has been enlarged by such work as putting dwellings into sanitary order, sometimes even paying the rent of suitable dwellings, and sending whole families into specially constructed dwellings in the country when the state of the patients warrants hope of a cure. The list of employments of the patients who have sought the aid of the dispensary is instructive. At the top of the list come those employed in textile industries (66) and in the manufacture of clothes (55), while at the other end of the list are those whose occupation keeps them more or less in the open air, such as builders' workmen (7) and agricultural labourers (6).

## Figures and Tables

**Figure f1:**